# Expression of miRNA-29c in the carotid plaque and its association with diabetic mellitus

**DOI:** 10.3389/fcvm.2024.1276066

**Published:** 2024-02-05

**Authors:** Hua Wang, Peipei Mai, Fang He, Yanfang Zhang

**Affiliations:** ^1^ Division of Graduate, Xinxiang Medical University, Xinxiang, Henan, China; ^2^ Department of Ultrasonography, Luoyang Central Hospital Affiliated to Zhengzhou University, Luoyang, China; ^3^ Department of Endocrinology, Luoyang Central Hospital Affiliated to Zhengzhou University, Luoyang, China

**Keywords:** miRNA-29c, plaque vulnerability, diabetic mellitus, vascular smooth muscle cells, VSMC

## Abstract

**Background:**

Carotid artery atherosclerosis is a major cause of ischemic stroke, and ischemic stroke is the leading cause of morbidity and mortality worldwide. Unfortunately, the reason for the build-up of atherosclerosis plaque is unknown. The miRNA-29c was reported to promote the phenotype transformation of vascular smooth muscle cells (VSMCs) in diabetes mice, eventually leading to plaque formation and bleeding. However, such studies are rare and limited to animal experiments.

**Methods:**

In our study, 40 patients were divided into a diabetic mellitus (DM) group and a non-DM group according to whether they were diagnosed with DM. Then, the real-time quantitative PCR was applied to examine the miRNA-29c level in human carotid plaque tissue derived from 40 subjects receiving carotid endarterectomy.

**Results:**

Briefly, diabetes patients had a decreased miRNA-29c level as compared with non-DM subjects, and this comparison was statistically significant (*P *= 0.02). Notably, variable miRNA-29c level was negatively associated with HbA1c level, although no statistical significance was observed. Moreover, there was an increased miRNA-29c level in patients with cerebral stroke.

**Conclusion:**

Collectively, the miRNA-29c level in the carotid plaque is closely associated with DM and cerebral stroke, which may contribute to atherosclerosis formation.

## Introduction

Atherosclerosis in carotid arteries is an important contributor to ischemic cerebral stroke. In addition to the atherosclerotic plaque size, plaque vulnerability is a key determinant of the likelihood that a plaque induces cerebral stroke (1, 2). Studies have identified that phenotypic transformation and proliferation of vascular smooth muscle cells (VSMCs) are the key factors to promote the vulnerability of carotid atherosclerotic plaque (3–6). Shankman et al. demonstrated that AMPK*α*2 deletion induced VSMC phenotypic transition and promoted atherosclerotic plaque instability in a nuclear factor-κB-KLF4 dependent manner (4). Bennett et al. believed that a full understanding of VSMC behavior in atherosclerosis is critical to identifying the therapeutic targets for both prevention and treatment of atherosclerosis (6). They argued that VSMC proliferation may be beneficial throughout atherosclerosis, not just in advanced lesions; moreover, VSMC apoptosis, cell senescence, and VSMC-derived macrophage-like cells may promote inflammation. Research has shown that the proliferation rate of VSMCs in diabetes patients is nearly 50% higher than that in non-DM patients. Especially, diabetes patients exhibit a high VSMC phenotype transformation, which may contribute to the high incidence of vascular complications (7–11). How to identify and stabilize vulnerable plaques is of great value in patients at risk of lesion rupture. However, the reliable test for assessing plaque vulnerability is rare up to now. Therefore, there is an urgent need to develop reliable biomarkers for the detection and prediction of high-risk plaques.

MiRNAs are small non-coding RNAs (22-nucleotides long) that regulate several biological processes by negatively regulating their target genes via post-transcriptional mechanisms. Dysregulation of miRNAs expression has been implicated in several pathophysiological conditions including vascular disease (12–14). VSMC-specific deletion of Dicer, an RNase III endonuclease essential for the biogenesis of miRNAs, is associated with internal hemorrhage, defective blood vessels, reduced VSMC proliferation and contractile phenotype, suggesting important functions for miRNAs in VSMC during development. Furthermore, Dicer knock-down in adult mice reduced global miRNA levels and blood pressure, suggesting the critical role of miRNA in VSMC functions (15, 16). Patients with diabetic mellitus (DM) exhibit significant deregulation of miRNAs involved in angiogenesis, vascular repair, and endothelial homeostasis (17). The VSMC phenotype is important in maintaining tissue elasticity, wall stress homeostasis and vessel stiffness. In vascular proliferative disorders, VSMC phenotypic switch is a key cellular event in arterial remodeling (18, 19). The VSMCs exhibit increased proliferation, adhesion, and migration in diabetes compared with non-DM patients (7, 9, 11, 20). Recently, a large number of animal studies demonstrated that miRNAs play a key role in VSMC functions, and VSMC phenotypic switch commences with cell de-differentiation through down-regulation of VSMC contractile genes (21). The miRNA-29c is specifically expressed by contractile VSMCs and its down-regulation is classically related to the phenotypic switch of VSMCs underlying vascular proliferative disorders (21, 22). MiRNA-29c involves in VSMC proliferation through repression of Emp2 in DM rats (23). However, there are few studies on the expression level of miRNA-29c in human carotid plaques.

In the present study, the miRNA-29c level in a cohort of carotid endarterectomy plaque tissues was investigated using a real-time quantitative PCR approach. We preliminarily explored the difference for the clinicopathological characteristics of patients to determine whether miRNA-29c could be used as a biomarker of unstable plaque/plaque rupture in DM patients.

## Materials and methods

### Patients and tissue samples

This study obtained the approval of our institutional review board and human ethics committee. From January 2020 to July 2023, 40 paraffin-embedded carotid plaque tissues were selected randomly at the Luoyang Central Hospital Affiliated to Zhengzhou University. Clinicopathological data were acquired from the patients' files or by interview with the patients or their relatives, and these data were summarized in Table 1.

**Table 1:** The general clinicopathological characteristics in 40 patients.

Characteristics	No. of patients (%)
Gender	
Male	30 (75)
Female	10 (25)
Age, years	
L	12 (30)
H	28 (60)
Smoking history	
Yes	22 (55)
No	18 (45)
Drinking history	
Yes	33
No	7
BMI	
≤24	12
>24	28
FBG	
<7.0	27
>7.0	13
HDL	
≤1.0	20
>1.0	20
LDL	
≤2.1	20
>2.1	20
TC	
≤4.1	20
>4.1	20
TG	
≤1.3	20
>1.3	20
CHD	
Yes	13
No	27
Diabetes	
Yes	25
No	15
Cerebral stroke	
Yes	22
No	18
Hypertension	
Yes	13
No	27

BMI, body mass index; FBG, fasting blood glucose; HDL, high density lipoprotein; LDL, low density lipoprotein; TC, total cholesterol; TG, triglyceride; CHD, coronary atherosclerotic heart disease.

### Preparation of cDNA

RNA was extracted from paraffin-embedded tissues. Serial sections were obtained from each sample. Briefly, the tissues were first treated with xylene for 20 min at room temperature to remove the paraffin, then the total RNA from tissue specimens was isolated by a DP502 reagent kit following manufacturer's instruction (TIANGEN Inc., Beijing, P.R. China). Next, the cDNA was prepared using kr211 reagent kit (TIANGEN Inc., Beijing, P.R. China), and stored at −80 °C until used.

### Analysis of miRNA-29c expression level

A real-time quantitative PCR was used to measure the relative expression level of miRNA-29c in the samples of DM and control subjects. To be specific, real-time quantitative PCR was carried out using the reagents and protocol of the SYBR Green qPCR Master Mix (FP411, TIANGEN Inc., Beijing, P.R. China). MiRNA-29c and U6 primer sequences were purchased from TIANGEN company (CD201-0332, CD201-0145, TIANGEN Inc., Beijing, P.R. China). Briefly, in the first step, the template was denatured at 95 °C for 5 min. The second step consisted of 45 cycles at 94 °C for 20 s and 60 °C for 34 s. Each sample was run in triplicate. In this study, the 2^−ΔΔ*Ct*^ method was applied to quantify. U6 was used as a loading control to normalize the expression level of the target protein.

### Statistical analysis

The Mann–Whitney *U*-test was used to compare the expression level of miRNA-29c, and SPSS statistical package (version 16.0, Chicago, IL, USA) to evaluate the association between the expression level of miRNA-29c and different clinical features. Then, a multivariate model was developed to adjust the most important covariates, including gender, age, drinking, smoking and hypertension. SPSS statistical package (version 16.0, Chicago, IL) was used for all statistical analysis. *P* < 0.05 indicated a significant difference.

## Results

### Relative miRNA-29c expression fold change in general clinical characteristics

**Figure 1:**
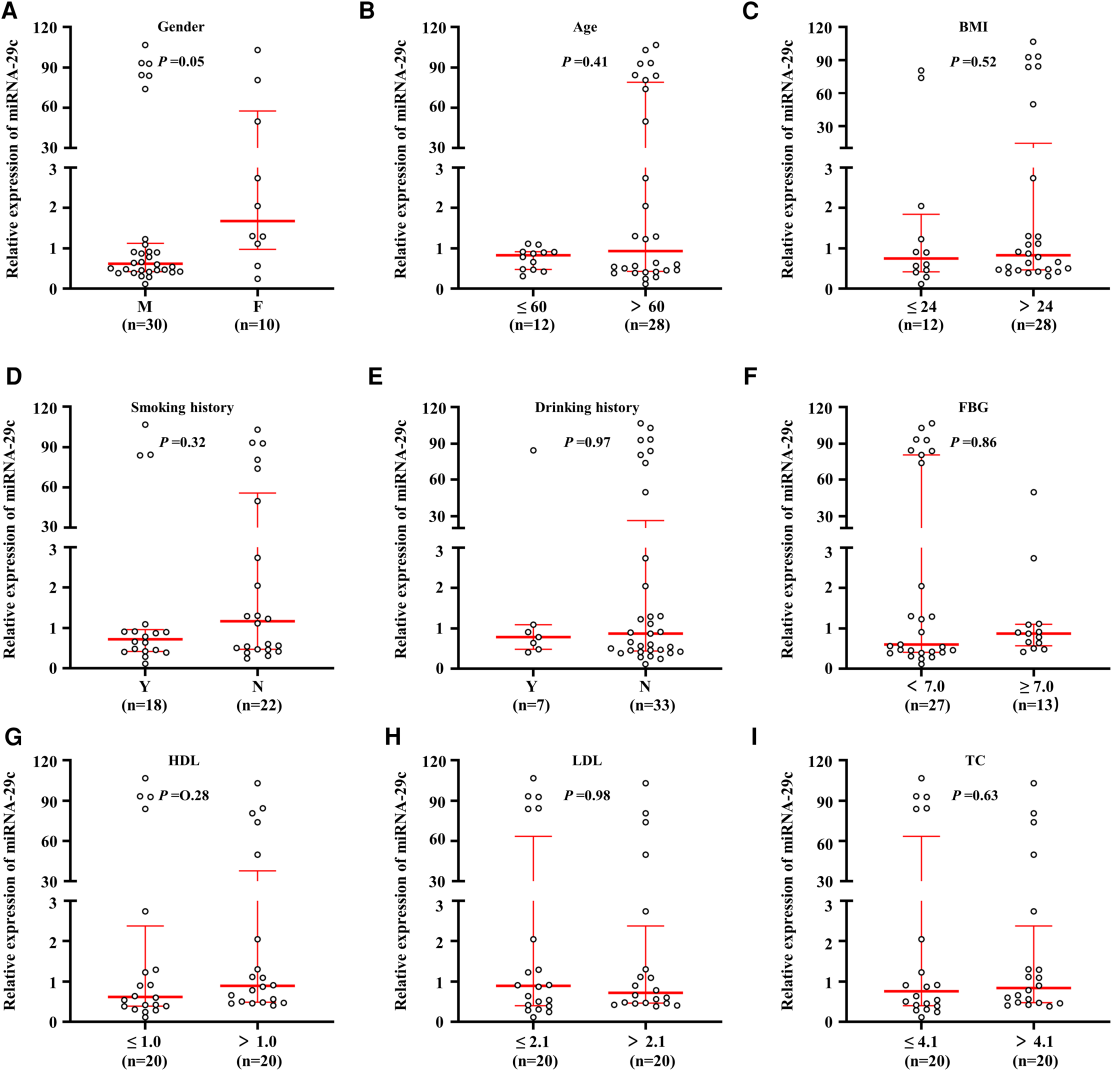
Association of miRNA-29c level with clinical characteristics in carotid plaques tissues. MiRNA-29c level was analyzed using real-time quantitative PCR approach. Correlation analysis of miRNA-29c expression levels with Gender (**A**), Age (**B**), BMI (**C**), Smoking history (**D**), Drinking history (**E**), FBG (**F**), HDL (**G**), LDL (**H**), and TC (**I**). The circle represented the miRNA-29c level of each case. The measurement data was divided into two groups based on the median. The horizontal lines represented median ± interquartile range. The sample medians were compared using the Mann-Whitney *U* test. BMI, body mass index; FBG, fasting blood glucose; HDL, high density lipoprotein; LDL, low density lipoprotein; TC, total cholesterol; TG, triglyceride; CHD, coronary atherosclerotic heart disease; M, male; F, female; N, No; Y, Yes.

Real-time quantitative PCR assay was performed to analyze the miRNA-29c level in 40 carotid plaque specimens. We evaluated whether miRNA-29c level differed by the selected general clinicopathological characteristics. These general clinical characteristics included gender (male, *n* = 30; female, *n* = 10), age, smoking history (yes, *n* = 18; no, *n* = 22), drinking history (yes, *n* = 7; no, *n* = 33), body mass index (BMI), fasting blood glucose (FBG), high density lipoprotein (HDL), low density lipoprotein (LDL), total cholesterol (TC), triglyceride (TG), coronary atherosclerotic heart disease (CHD), diabetes, cerebral stroke, and hypertension. According to the international standards, the indicators used to judge whether there was hypertension, stroke, coronary heart disease and diabetes, 60 years old was used as the cutoff value of age (≤60, *n* = 12; >60, *n* = 28), 24 as the cutoff value of Body mass index (BMI: ≤24, *n* = 12; >24, *n* = 28), 7.0 as the cutoff value of FBG (<7.0, *n* = 27; >7.0, *n* = 13), HDL median 1.0 as the cutoff value (≤1.0, *n* = 20; >1.0, *n* = 20); LDL median 2.1 as the cutoff value (≤2.1, *n* = 20; >2.1, *n* = 20); TC median 4.1 as the cutoff value (≤4.1, *n* = 20; >4.1, *n* = 20); TG median 1.3 as the cutoff value (≤1.3, *n* = 20; >1.3, *n* = 20) (Table 1). As shown in Figure 1, the median expression fold change of miRNA-29c in female patients with 1.68 (range = 0.97–57.59) was significantly higher than those with 0.62 (range = 0.42–1.13) (*P* = 0.05). Considering the limited cases included in this study, further verification was required. Notably, although no statistical significance was noted, non-smoking patients had a higher miRNA-29c expression fold change as compared with smoking patients (*P *= 0.32); high HDL patients had a higher miRNA-29c expression than low HDL cases (*P* = 0.28). We did not find significant associations of miRNA-29c expression fold change with age (*P* = 0.41), BMI (*P* = 0.52), drinking history (*P* = 0.97) FBG (*P* = 0.86) and TC (*P* = 0.63) (Figure 1).

### Relative miRNA-29c expression fold change in the diseases

**Figure 2:**
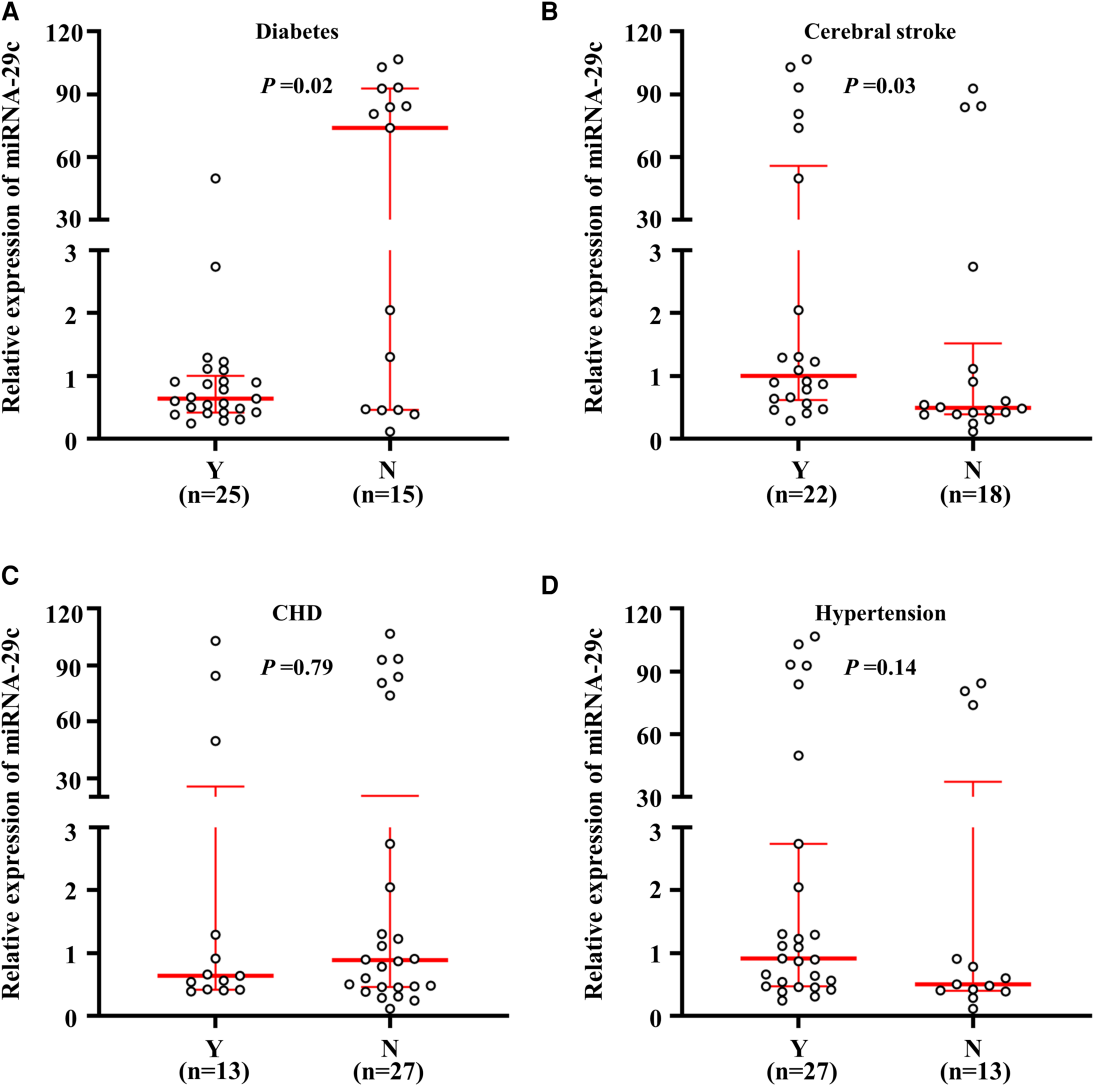
Relative miRNA-29c expression fold change in the diseases. A real-time quantitative PCR assay was performed to analyze the miRNA-29c level in subjects. MiRNA-29c level corresponding to each individual case of diabetes. and non-diabetes (**A**), cerebral stroke and non-cerebral stroke (**B**), CHD and non CHD (**C**), hypertension and non-hypertension carotid plaques tissues (**D**). The horizontal lines represented median ± interquartile range. N, No; Y, Yes.

Given the close relationship of carotid plaque with hypertension, stroke, coronary heart disease and diabetes, the miRNA-29c expression fold change in these diseases was analyzed next. As shown in Figure 2, the median value of miRNA-29c expression was higher in non-DM patients than in DM patients, and the difference was statistically significant (*P *= 0.02). The median value among DM patients and control subjects were 0.63 (range = 0.25–49.89) and 73.99 (range = 0.12–106.78), respectively, suggesting that the majority of DM patients had lower miRNA-29c expression as compared to control subjects. Additionally, the cerebral stroke group exhibited a higher miRNA-29c level compared with the non-cerebral stroke group [0.91 (range = 0.56–49.89) vs. 0.48 (range = 0.39–1.12) (*P* = 0.03)] (Figure 2). Besides, we did not find significant associations of miRNA-29c expression fold change with CHD (*P* = 0.79) and hypertension (*P* = 0.14) (Figure 2).

### Associations between variable miRNA-29c expression fold change and clinical characteristics in carotid plaque patients

Given frequently altered miRNA-29c expression fold change in carotid plaque, the associations between the miRNA-29c expression fold change and the clinicopathological characteristics were investigated. The outcomes of univariate analyses revealed that miRNA-29c expression fold change was decreased in patients with diabetes as compared with the non-DM patients (the correlation coefficient *r* = −0.374, *P *< 0.05) (Table 2). We were also surprised that the correlation between miRNA-29c and HbA1c was not statistically significant (the correlation coefficient *r* = −0.247, *P *> 0.05) but negatively correlated, indirectly verifying that the expression fold change of miRNA-29c in DM patients was significantly lower than that in non-DM patients (Table 2). Despite no statistical significance, miRNA-29c was positively correlated with age (*r* = 0.082, *P* = 0.614), BMI (*r* = 0.052, *P* = 0.759), HDL (*r* = 0.177, *P* = 0.276), TC (*r* = 0.076, *P* = 0.643), and hypertension (*r* = 0.234, *P* = 0.147). Furthermore, miRNA-29c was negatively associated with gender (*r* = −0.310, *P* = 0.051), LDL (*r* = −0.056, *P* = 0.731), drinking history (*r* = −0.004, *P* = 0.981), smoking history (*r* = −0.161, *P *= 0.321), CHD (*r* = −0.034, *P* = 0.838) (Table 2).

**Table 2:** Correlation analysis of group and miRNA-29c.

	Group	MiR-29c	Gender	Age	BMI	LDL	HDL	TC	TG	HbA1c	Drinking history	Smoking history	CHD	Hypertension	Cerebral Stroke
Group	1.000	−.374*	.030	−.164	.057	.125	−.054	.101	.098	.835**	.224	.078	.207	.014	−.078
MiR-29c		1.000	−.310	.082	.052	−.056	.177	.076	.162	−.247	−.004	−.161	−.034	.234	.335*
Gender			1.000	−.161	.170	−.198	−.235	−.345*	−.085	−.063	.265	.522**	−.092	−.277	−.174
Age				1.000	−.017	−.263	−.282	−.278	−.158	−.170	−.347*	−.469**	.021	.172	−.146
BMI					1.000	−.032	−.335*	−.098	.271	.099	−.051	−.027	.173	.341*	−.199
L-HDL						1.000	.316*	.937**	.186	.200	.075	−.067	−.028	−.097	.120
H-HDL							1.000	.460**	−.153	.029	.243	−.017	−.051	−.217	.200
TC								1.000	.282	.196	.085	−.170	.012	−.113	.213
TG									1.000	.085	.118	−.057	.090	−.072	.163
HbA1c										1.000	.149	.085	.090	.160	−.026
Drinking history											1.000	.508**	.091	−.364*	.030
Smoking history												1.000	.123	−.338*	.010
CHD													1.000	.026	.091
Hypertension														1.000	.231
Cerebral Stroke															1.000

*P < 0.05.

**P < 0.05.

## Discussion

The rupture and hemorrhage of vulnerable plaque in carotid artery are the main causes of ischemic stroke. Notably, VSMC proliferation and migration are the important mechanisms of rupture and hemorrhage of plaque. The normal adult artery is mainly composed of VSMCs. VSMCs are differentiated cells in a low secretory state. The main function of VSMCs is to maintain the elasticity and contraction of blood vessels (7). VSMCs have poor or no ability for proliferation and migration, fusiform or banded cell bodies, a large number of myofilaments and structural proteins, less content of synthetic organelles such as rough endoplasmic reticulum and Golgi complex, poor or no ability for matrix synthesis, and small volume (24). Secretory VSMCs mainly exist in the blood vessels and pathological vessels in the middle stage of embryo, with the main function of proliferation, migration into the intima and extracellular matrix protein synthesis. Secretory VSMCs are similar to fibroblasts in morphology, with less content of myofilament and structural protein, more synthetic organelles, stronger ability to synthesize and secrete matrix protein, and larger volume than contractile VSMC (24). The synthetic type of VSMC has strong secretory ability, can proliferate and migrate in large quantity, and produce extracellular matrix protein, leading to functional disorder, thickening of tube wall, narrowing of lumen, reduction of vascular compliance and vascular remodeling (24). Phenotypic transformation is the premise of VSMC proliferation and migration. VSMC phenotypic switch is a key cellular event in vasculo-proliferative disorders (18, 19), and VSMCs display a different and more aggressive phenotype in DM patients (11, 20, 25). DM patients exhibit significant deregulation of miRNAs involved in angiogenesis, vascular repair, and endothelial homeostasis (17). The diabetic detrimental hyperplastic phenotypes of VSMCs are characterized by a microRNA signature that diverges significantly from the microRNA network activated in non-DM VSMCs. For example, some studies have found that miRNA-29c can regulate the phenotypic transformation of VSMCs through Emp2 (23). However, the number of cases in this study is small, and the persuasion is not strong, which cannot truly and effectively reflect the experimental results. At present, there is no systematic report on the value of miRNA-29c expression level in the diagnosis of carotid plaque vulnerability in DM and non-DM patients.

In this study, a real-time quantitative PCR was performed to assess the relative miRNA-29c level in a cohort of carotid plaque tissues. To further explore the relationships between the miRNA-29c level and the clinicopathological characteristics, the patients were assigned into two groups according to the gender, age, drinking history, smoking history, BMI, FBG, HDL, LDL and TC, respectively. It was worth noting that, the female patients had a higher miRNA-29c level than male cases, and the increased miRNA-29c was positively associated with HDL level, although the difference was not statistically significant (*P *= 0.05).

Given a close correlation of carotid plaque with diabetes, hypertention, cerebral stroke and CHD, the association of the miRNA-29c level with these diseases was further explored. It was surprised that the miRNA-29c level was significantly lower in DM patients than in non-DM ones, which was consistent with previous animal studies (23). Taken together, it is reasonable to hypothesize that decreased miRNA29c may contribute to the VSMC phenotypic formation in DM patient. Notably, we determined that cerebral stroke patients had increased miRNA29c level relative to on-cerebral ones. Therefore, we speculate that miRNA-29c level may play a key role in carotid plaque vulnerability. Previous research finding revealed that the down-regulation of miRNA-29c may not only cause phenotype transformation and dysfunction of VSMC (23), but also contribute to the carotid plaque build-up, rupture and bleed in DM patients (23). The above results indicate that the variable expression level of miRNA-29c may be helpful for accurately guiding the early diagnosis and treatment of stroke in clinical practice.

However, there were only 40 cases included in this study, and more subjects needed to be collected in the following study to verify these results. In addition, relevant experiments *in vitro* and *in vivo* are also is necessary to further elucidate the mechanism of miRNA-29c in the formation of VSMC phenotype in DM patients.

## Conclusion

To sum up, we studied the miRNA-29c level in a cohort of carotid plaque specimens and discovered a close correlation between the expression level of miRNA-29c and DM-related complications. The expression level of miRNA-29c may contribute to atherosclerosis build-up and serve as a marker in the evaluation of plaque vulnerability and cerebral stroke.

## Data Availability

The original contributions presented in the study are included in the article/Supplementary Material, further inquiries can be directed to the corresponding author.

## References

[1] Shah PK. Mechanisms of plaque vulnerability and rupture. J Am Coll Cardiol. (2003) 41:15S–22S. 10.1016/s0735-1097(02)02834-6 12644336

[2] Saba L Potters F van der Lugt A Mallarini G. Imaging of the fibrous cap in atherosclerotic carotid plaque. Cardiovasc Intervent Radiol. (2010) 33:681–9. 10.1007/s00270-010-9828-8 20237780

[3] Ding Y Zhang M Zhang W Lu Q Cai Z Song P AMP-activated protein kinase alpha 2 deletion induces VSMC phenotypic switching and reduces features of atherosclerotic plaque stability. Circ Res. (2016) 119:718–30. 10.1161/CIRCRESAHA.116.308689 27439892 PMC6265658

[4] Shankman LS Gomez D Cherepanova OA Salmon M Alencar GF Haskins RM KLF4-dependent phenotypic modulation of smooth muscle cells has a key role in atherosclerotic plaque pathogenesis. Nat Med. (2015) 21:628–37. 10.1038/nm.3866 25985364 PMC4552085

[5] Horita H Wysoczynski CL Walker LA Moulton KS Li M Ostriker A Nuclear PTEN functions as an essential regulator of SRF-dependent transcription to control smooth muscle differentiation. Nat Commun. (2016) 7:10830. 10.1038/ncomms10830 26940659 PMC5411712

[6] Bennett MR Sinha S Owens GK. Vascular smooth muscle cells in atherosclerosis. Circ Res. (2016) 118:692–702. 10.1161/CIRCRESAHA.115.306361 26892967 PMC4762053

[7] Faries PL Rohan DI Takahara H Wyers MC Contreras MA Quist WC Human vascular smooth muscle cells of diabetic origin exhibit increased proliferation, adhesion, and migration. J Vasc Surg. (2001) 33:601–7. 10.1067/mva.2001.111806 11241133

[8] Porter KE Riches K. The vascular smooth muscle cell: a therapeutic target in type 2 diabetes? Clin Sci (Lond). (2013) 125:167–82. 10.1042/CS20120413 23634936

[9] Zhang X Liu L Chen C Chi YL Yang XQ Xu Y Interferon regulatory factor-1 together with reactive oxygen species promotes the acceleration of cell cycle progression by up-regulating the cyclin E and CDK2 genes during high glucose-induced proliferation of vascular smooth muscle cells. Cardiovasc Diabetol. (2013) 12:147. 10.1186/1475-2840-12-147 24119616 PMC3852693

[10] Madi HA Riches K Warburton P O'Regan DJ Turner NA Porter KE. Inherent differences in morphology, proliferation, and migration in saphenous vein smooth muscle cells cultured from nondiabetic and type 2 diabetic patients. Am J Physiol Cell Physiol. (2009) 297:C1307–17. 10.1152/ajpcell.00608.2008 19741193

[11] Engberding N San Martin A Martin-Garrido A Koga M Pounkova L Lyons E Insulin-like growth factor-1 receptor expression masks the antiinflammatory and glucose uptake capacity of insulin in vascular smooth muscle cells. Arterioscler Thromb Vasc Biol. (2009) 29:408–15. 10.1161/ATVBAHA.108.181727 19122171 PMC2713108

[12] Maegdefessel L Rayner KJ Leeper NJ. MicroRNA regulation of vascular smooth muscle function and phenotype: early career committee contribution. Arterioscler Thromb Vasc Biol. (2015) 35:2–6. 10.1161/ATVBAHA.114.304877 25520518

[13] Mendell JT Olson EN. MicroRNAs in stress signaling and human disease. Cell. (2012) 148:1172–87. 10.1016/j.cell.2012.02.005 22424228 PMC3308137

[14] Urbich C Kuehbacher A Dimmeler S. Role of microRNAs in vascular diseases, inflammation, and angiogenesis. Cardiovasc Res. (2008) 79:581–8. 10.1093/cvr/cvn156 18550634

[15] Albinsson S Suarez Y Skoura A Offermanns S Miano JM Sessa WC. MicroRNAs are necessary for vascular smooth muscle growth, differentiation, and function. Arterioscler Thromb Vasc Biol. (2010) 30:1118–26. 10.1161/ATVBAHA.109.200873 20378849 PMC2880481

[16] Albinsson S Skoura A Yu J DiLorenzo A Fernandez-Hernando C Offermanns S Smooth muscle miRNAs are critical for post-natal regulation of blood pressure and vascular function. PLoS One. (2011) 6:e18869. 10.1371/journal.pone.0018869 21526127 PMC3081311

[17] Paneni F Beckman JA Creager MA Cosentino F. Diabetes and vascular disease: pathophysiology, clinical consequences, and medical therapy: part I. Eur Heart J. (2013) 34:2436–43. 10.1093/eurheartj/eht149 23625211

[18] Torella D Gasparri C Ellison GM Curcio A Leone A Vicinanza C Differential regulation of vascular smooth muscle and endothelial cell proliferation in vitro and in vivo by cAMP/PKA-activated p85alphaPI3K. Am J Physiol Heart Circ Physiol. (2009) 297:H2015–25. 10.1152/ajpheart.00738.2009 19783773

[19] Nguyen AT Gomez D Bell RD Campbell JH Clowes AW Gabbiani G Smooth muscle cell plasticity: fact or fiction? Circ Res. (2013) 112:17–22. 10.1161/CIRCRESAHA.112.281048 23093573 PMC4135725

[20] Wang CC Gurevich I Draznin B. Insulin affects vascular smooth muscle cell phenotype and migration via distinct signaling pathways. Diabetes. (2003) 52:2562–9. 10.2337/diabetes.52.10.2562 14514641

[21] Torella D Iaconetti C Catalucci D Ellison GM Leone A Waring CD MicroRNA-133 controls vascular smooth muscle cell phenotypic switch in vitro and vascular remodeling in vivo. Circ Res. (2011) 109:880–93. 10.1161/CIRCRESAHA.111.240150 21852550

[22] Iaconetti C De Rosa S Polimeni A Sorrentino S Gareri C Carino A Down-regulation of miR-23b induces phenotypic switching of vascular smooth muscle cells in vitro and in vivo. Cardiovasc Res. (2015) 107:522–33. 10.1093/cvr/cvv141 25994172

[23] Torella D Iaconetti C Tarallo R Marino F Giurato G Veneziano C miRNA regulation of the hyperproliferative phenotype of vascular smooth muscle cells in diabetes. Diabetes. (2018) 67:2554–68. 10.2337/db17-1434 30257973

[24] Frismantiene A Philippova M Erne P Resink TJ. Smooth muscle cell-driven vascular diseases and molecular mechanisms of VSMC plasticity. Cell Signal. (2018) 52:48–64. 10.1016/j.cellsig.2018.08.019 30172025

[25] Grootaert MOJ Bennett MR. Vascular smooth muscle cells in atherosclerosis: time for a re-assessment. Cardiovasc Res. (2021) 117:2326–39. 10.1093/cvr/cvab046 33576407 PMC8479803

